# A Harmful Algal Bloom of *Karenia brevis* in the Northeastern Gulf of Mexico as Revealed by MODIS and VIIRS: A Comparison

**DOI:** 10.3390/s150202873

**Published:** 2015-01-28

**Authors:** Chuanmin Hu, Brian B. Barnes, Lin Qi, Alina A. Corcoran

**Affiliations:** 1 College of Marine Science, University of South Florida, St. Petersburg, FL 33701, USA; E-Mails: bbarnes4@mail.usf.edu (B.B.B.); lqi@mail.usf.edu (L.Q.); 2 Florida Fish and Wildlife Conservation Commission, Fish and Wildlife Research Institute, St. Petersburg, FL 33701, USA; E-Mail: alina.corcoran@MyFWC.com

**Keywords:** MODIS, VIIRS, fluorescence, chlorophyll *a*, harmful algal bloom, *Karenia brevis*, CDOM, Gulf of Mexico

## Abstract

The most recent Visible Infrared Imager Radiometer Suite (VIIRS) is not equipped with a spectral band to detect solar-stimulated phytoplankton fluorescence. The lack of such a band may affect the ability of VIIRS to detect and quantify harmful algal blooms (HABs) in coastal waters rich in colored dissolved organic matter (CDOM) because of the overlap of CDOM and chlorophyll absorption within the blue-green spectrum. A recent HAB dominated by the toxin-producing dinoflagellate *Karenia brevis* in the northeastern Gulf of Mexico, offshore of Florida's Big Bend region, allowed for comparison of the capacities of VIIRS and Moderate Resolution Imaging Spectroradiometer (MODIS) to detect blooms in CDOM-rich waters. Both VIIRS and MODIS showed general consistency in mapping the CDOM-rich dark water, which measured a maximum area of 8900 km^2^ by mid-July 2014. However, within the dark water, only MODIS allowed detection of bloom patches—as indicated by high normalized fluorescence line height (nFLH). Field surveys between late July and mid-September confirmed *Karenia brevis* at bloom abundances up to 20 million cells·L^−1^ within these patches. The bloom patches were well captured by the MODIS nFLH images, but not by the default chlorophyll *a* concentration (Chla) images from either MODIS or VIIRS. Spectral analysis showed that VIIRS could not discriminate these high-phytoplankton water patches within the dark water due to its lack of fluorescence band. Such a deficiency may be overcome with new algorithms or future satellite missions such as the U.S. NASA's Pre-Aerosol-Clouds-Ecology mission and the European Space Agency's Sentinel-3 mission.

## 1. Introduction

The most recent (2011–present) ocean color satellite sensor, the Visible Infrared Imager Radiometer Suite (VIIRS), is expected to provide a consistent, long-term data record to continue observations from its predecessors, including the Moderate Resolution Imaging Spectroradiometer (MODIS, 1999–present on Terra and 2002–present on Aqua) and the Medium Resolution Imaging Spectrometer (MERIS, 2002–2012). Achieving such a goal requires substantial effort in sensor calibration, algorithm development, and data product validation. Recent sensor evaluation in 2014 showed signs of sensor degradation in the near-infrared (NIR) bands and radiometric calibration in other bands (Menghua Wang of NOAA NESDIS, and Bryan Franz of NASA GSFC, personal communications). These artifacts are minimized in data re-processing by both NASA and NOAA, at least to the first order. For the global ocean, Wang *et al.* [1] showed general consistency between VIIRS and MODIS/Aqua measurements after proper vicarious calibration of VIIRS. For a coastal region, Hlaing *et al.* [2] showed general agreement between VIIRS products and field measurements. Comparison between the diffuse attenuation coefficient (*K*_d_490, m^−1^) data product for the Gulf of Mexico (GOM) showed consistency between VIIRS and MODIS/Aqua for both 2012 and 2013 [3]. Similarly, for a moderately turbid estuary (turbidity generally between 1 and 8 NTU, Tampa Bay, FL, USA), consistency was achieved between VIIRS and MODIS/Aqua for the remote sensing reflectance (Rrs, sr^−1^), chlorophyll *a* (Chla, mg m^−3^), and colored dissolved organic matter (CDOM) absorption (*a*_g_, m^−1^) data products for 2012 [4]. It is expected that VIIRS may continue the MODIS-quality data series with continuous improvement in sensor calibration and algorithm development.

Despite its potential to perpetuate MODIS-quality data, VIIRS has a considerable shortcoming. Unlike MODIS, VIIRS is not equipped with a spectral band in the red to detect solar stimulated chlorophyll fluorescence (Table 1). For most ocean waters, Chla can be estimated without this band using either empirical or semi-analytical approaches [5]. However, the lack of such a band may significantly hinder VIIRS capacity to study phytoplankton physiology in the global open ocean as revealed by MODIS [6] and to map phytoplankton blooms in CDOM-rich coastal waters, which present a considerable challenge as the absorption properties of CDOM in the blue-green spectral region are similar to those of phytoplankton pigments. Hu *et al.* [7] showed that MODIS fluorescence line height (FLH, [8]) data provided unique information to differentiate a phytoplankton bloom in CDOM-rich waters off southwest Florida, which was confirmed by field measurements to contain high concentrations of the toxic dinoflagellate, *Karenia brevis*, the phytoplankton species responsible for most of Florida's harmful algal blooms (HABs). The capability of the MODIS FLH product is possible because the 678-nm MODIS band is more sensitive to chlorophyll fluorescence than it is to CDOM. Indeed, McKee *et al.* [9] and Gilerson *et al.* [10] found that although FLH is sensitive to perturbations by non-living particles (e.g., suspended sediments), it is relatively insensitive to CDOM perturbations: a 20-fold increase in CDOM would lead to only 50% decrease in FLH. Then, for *K. brevis* HAB detection, a two-step approach can be used: a phytoplankton bloom is first identified using MODIS FLH data; then, field measurements can confirm whether such a bloom is due to *K. brevis* or other non-harmful phytoplankton species (e.g., diatoms).

There are several other ways instead of using MODIS FLH to detect and monitor *K. brevis* HABs in the GOM [11–15]. While they differ in band selection and algorithm design, the basic principle is the same: a bloom is first detected using either traditional algorithms involving the blue/green bands or the NIR/red bands, and then the bloom type (*K. brevis* or other) is determined through examining either the backscattering efficiency [12], spectral curvature [13], or normalized water leaving radiance (nLw) in the green [15]. Soto-Ramos [16] performed a thorough review of these approaches including the FLH method, and concluded that they all performed similarly with different strengths and weaknesses for different conditions.

**Figure 1.**
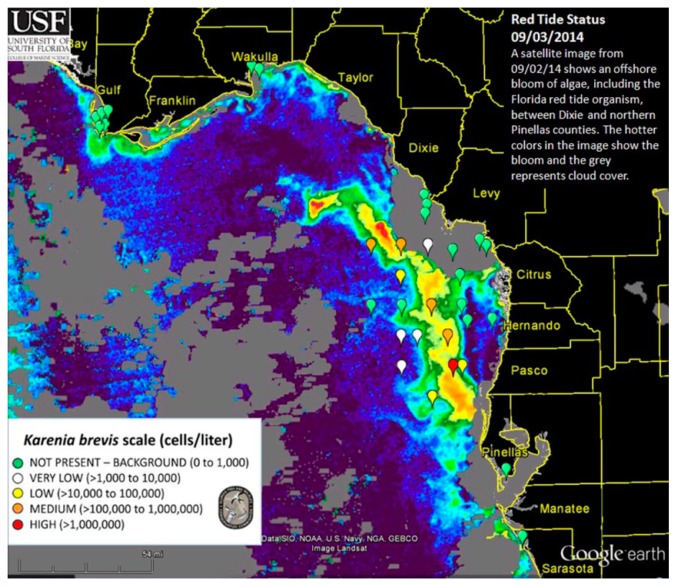
A sample HAB bulletin created and distributed weekly or twice weekly (MyFWC.com/RedTideStatus) by the Florida Fish and Wildlife Conservation Commission (FWC), the official agency of the state of Florida responsible for red tide monitoring. The background image is a MODIS normalized fluorescence line height (nFLH) image, annotated with *K. brevis* concentrations determined from water sample analysis. Interpretation is created by a human analyst and annotated on the image.

Currently, two approaches are being used operationally by the U.S. Federal and state agencies to detect and monitor *K. brevis* HABs in near real-time in the GOM. The NOAA HAB bulletin (http://tidesandcurrents.noaa.gov/hab/bulletins.html) uses the Chla-anomaly method proposed by [11] to first detect “new” bloom patches, and then analyzes the spectral curvature to determine whether they are *K. brevis* blooms [13]. The official agency of the state of Florida responsible for red tide monitoring, the Florida Fish and Wildlife Conservation Commission (FWC, Saint Petersburg, FL, USA), uses the normalized FLH (nFLH = FLH normalized to solar irradiance) imagery to detect bloom patches in dark waters [7], and then combines field measurements to infer whether they are *K. brevis* blooms. An example of the latter bulletins is presented in Figure 1. Both types of bulletins with are distributed routinely to various stakeholders through the Web, email subscriptions, and social media. The nFLH and other customized imagery, annotated with water sample analysis whenever they are available, are also available through a Virtual Antenna System (VAS) every day in near real-time [17].

In short, MODIS nFLH provides a unique product to delineate and quantify blooms in CDOM-rich waters. This capacity is enhanced by the Medium Resolution Imaging Spectrometer (MERIS, European Space Agency, Paris, France), which has an additional band at 709-nm to characterize intense blooms due to the red-edge reflectance [18]. Unfortunately, VIIRS is not equipped with either the 678-nm or the 709-nm band, possibly limiting its capacity in studying coastal blooms. Given that both MODIS sensors are aging, the question is whether VIIRS can continue MODIS observations of HABs in case the two MODIS sensors stop functioning. Obviously, without a band between 675 and 710 nm, VIIRS cannot provide nFLH-type imagery. However, whether such a deficiency limits its capacity to detect and monitor *K. brevis* blooms still needs to be evaluated. The objective of this work is therefore to use a HAB event in the northeastern GOM (NEGOM) to compare the performance of VIIRS and MODIS, with a particular emphasis on the fluorescence band.

## 2. Data and Methods

Both VIIRS and MODIS/Aqua data were obtained from the NASA Goddard Space Flight Center and processed using the VAS (Hu *et al.*, [4]). Specifically, the low-level data were processed using the most recent updates in calibration and sensor artifacts correction (reprocessing version R2013.0, http://oceancolor.gsfc.nasa.gov/WIKI/OCReproc.html, NASA Goddard Space Flight Center, Greenbelt, MD, USA) embedded in the SeaDAS (version 7.0, NASA GSFC, Greenbelt, MD, USA) software package. The level-2 data products included spectral Rrs(λ), nFLH (VIIRS does not have nFLH), Chla (from the blue/green band ratio algorithms [19]), and total non-water absorption coefficient (*a*_t_443, m^−1^) from the Quasi Analytical Algorithm [20] (QAA). For validation using field data, satellite data associated with the various quality control flags (l2_flags) were discarded as invalid data [3].

Vertical profiles of temperature, salinity, and Chla fluorescence (relative units) were obtained with an instrument package at several stations during an event response cruise on 23 July 2014. Between July and September water samples were also obtained from volunteers and from event-response cruises with a 2.2-L β sampling bottle (Wildco, Yulee, FL, USA). Subsamples of 250 mL were immediately preserved in Lugol's iodine [21] and were examined with light microscopy within 24h for identification and enumeration of HAB species under 200× magnification using either a Axiovert 100 (Zeiss, Thornwood, NY, USA) or DP70 (Olympus, Center Valley, PA, USA) inverted microscope [22]. For enumeration, three milliliters of each of the preserved samples were settled for at least 30 minutes in a Lab-Tek coverslip bottom chamber [23] (#155379, Nalgene-Nunc, Rochester, NY, USA). HAB species, defined by potential to produce toxins or by absolute biomass, in the entire chamber were identified to the lowest taxonomic level possible and enumerated.

## 3. Results and Discussion

### 3.1. Results

**Figure 2.**
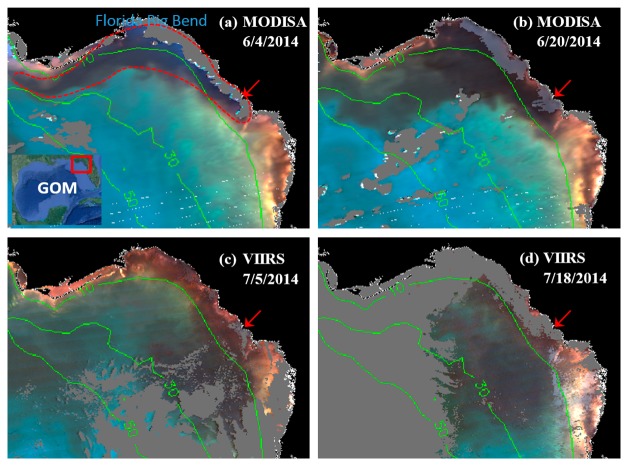
(**a**–**d**): MODIS and VIIRS ERGB images showing the progression of a dark water event off Florida's Big Bend region. The dark water in (**a**) is outlined approximately with the red dotted line. Annotated are the water depth contour lines in meters. The images cover 28°–30.2°N, 85.5°–82.5°W. The location of the Suwannee River mouth is annotated with red arrows. Gray represents clouds or invalid data.

**Figure 3.**
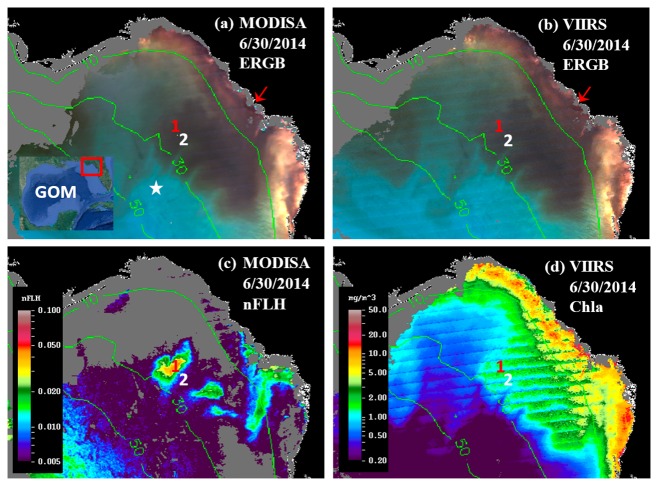
MODISA and VIIRS ERGB images on 30 June 2014 (19:00 GMT) in (**a**) and (**b**), respectively, showing dark water patches off the Suwannee River mouth (red arrows); (**c**) MODISA nFLH image (mW·cm^−2^·μm^−1^·sr^−1^) showing distinctive high-nFLH water patches in the dark water; (**d**) VIIRS Chla showing continuous high-Chla corresponding to the dark water. The image banding is due to sensor artifacts. Two locations (1° and 2°, 29.0534°N 83.9102°W and 28.9034°N 83.8420°W) corresponding to high- and low-nFLH values, respectively, were randomly selected and annotated on all images for further spectral analysis (Figure 5). The moderate nFLH values on the bottom left corner of (**c**) are due to sun glint contamination, which can be easily ruled out as non-blooms through visual inspection. The images cover 28°–30.2°N, 85.5°–82.5°W in the NEGOM region.

MODISA and VIIRS enhanced Red-Green-Blue (ERGB) imagery revealed a dark water event in June and July 2014. A sample sequence of the ERGB imagery can be found in Figure 2. The ERGB imagery was composed of the Rrs data at 547 (Red), 488 (Green), and 443 nm (Blue), and the dark color was due to the strong absorption at 443 and 488 nm by phytoplankton pigments and/or CDOM. Thus, if the dark water was associated with high FLH values, it must be a bloom (not necessarily *K. brevis* unless confirmed), and otherwise it must be a CDOM plume [7,24]. On June 4, dark water was observed from inshore to just past the 10-m isobath in Florida's Big Bend Region across a 7500 km^2^ area (red outline in Figure 2a). On June 30 (Figure 3a,b), the dark-water band separated from most of the shoreline, and the dark-water patch extended from the Suwannee River mouth to the southwest, reaching a surface area of ∼6400 km^2^ between the 10 and 30 m isobaths. By mid-July (Figure 2d), the dark-water patch, with a surface area of about 8900 km^2^, further extended to the west, between 10 and 50 m isobaths.

The MODISA and VIIRS data products on 30 June 2014 demonstrate similar capabilities in revealing the spatial distribution and intensity of dark water, as demonstrated by the nearly identical ERGB images (Figure 3a,b), suggesting that VIIRS could provide similar capacity as MODISA in delineating dark waters. For a more rigorous comparison, collocated MODISA and VIIRS Rrs data from June 30 (Figure 3a,b) were extracted from the 1-km pixels covering the entire study region after discarding data associated with the quality control flags.

**Figure 4.**
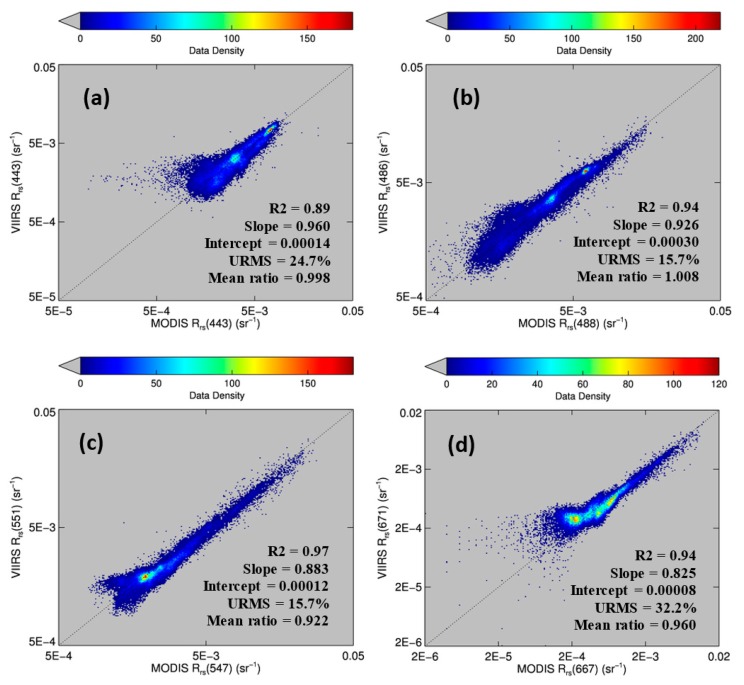
(**a**)–(**d**): Comparison between MODISA and VIIRS-derived Rrs data products. The data were collected in the NEGOM region on 30 June 2014 (Figure 3a,b). About 38,900 collocated data pairs from valid pixels on both MODISA and VIIRS were used to calculate the statistics. URMS is the unbiased RMS difference between the two measurements.

Figure 4 shows the comparison for four visible bands. Despite some data scattering, most of the pixels fell on the 1:1 line, indicating cross-sensor consistency. The mean ratios (VIIRS/MODISA) for the blue bands were close to 1.0. For the green and red bands, the mean ratios were 0.92 and 0.96, respectively, possibly due to the differences in the band centers (e.g., 547 nm for MODISA but 551 nm for VIIRS).

The offshore dark-water patches were delineated using a threshold of Rrs443 < 0.0026 sr^−1^. The threshold was determined from Rrs443 gradient images. In these images, the pixels separating the dark water from “non-dark” water had higher gradient values than the surrounding pixels. The mean Rrs443 value from these high-gradient pixels was about 0.0026 sr^−1^. For such determined dark-water pixels, there was more data scattering than for the brighter-water pixels (Figure 4), possibly due to atmospheric correction artifacts. Nevertheless, the Rrs comparison statistics indicated general agreement between MODISA and VIIRS in delineating dark waters. This observation is in line with those from a more comprehensive analysis to evaluate cross-sensor consistency [3]. The QAA-derived *a*_t_443 ranged from 0.08 to 0.21 m^−1^ in the dark water patch and from 0.025 to 0.05 m^−1^ in the nearby clear water (Figure 3a). Analysis of imagery from other dates showed similar results. However, without the fluorescence band(s), it was difficult to tell whether the dark water was caused by phytoplankton pigments, CDOM, or both. The availability of the MODIS 678-nm band made this puzzle easy to solve. Indeed, the MODIS nFLH image clearly showed several distinctive high-nFLH patches within the extensive dark water (Figure 3c). The area of the combined high-nFLH patches was estimated to be ∼3200 km^2^, only half the dark-water size between 10 and 50-m isobaths. In contrast, VIIRS could only show the blue/green band ratio Chla of about the same size as the dark water (Figure 3d).

**Figure 5.**
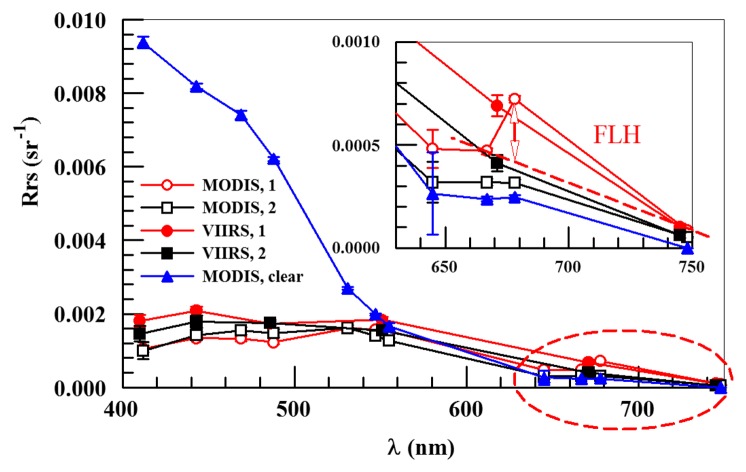
Rrs spectra from three locations of the NEGOM region (locations shown in Figure 3). Location 1 is in the high-nFLH bloom (circles) and Location 2 is in the nearby low-nFLH water (squares). For reference, MODIS Rrs from a clear-water location (annotated as a star in Figure 3a) is also shown. The spectra in the red and NIR are enlarged in the inset figure, where the calculation of nFLH for Location 1 is illustrated with a double-headed arrow. The standard error bars were derived from 3 × 3 1-km pixels centered at the locations. Note that although cross-sensor difference is relatively large for the blue bands, cross-location difference for the same sensor is much smaller.

The VIIRS Chla image did not reveal the spatial contrast of the MODIS nFLH image because the blue-green reflectance was rather similar across the entire dark-water region, as demonstrated in Figure 5. For the two randomly selected locations annotated as “1” and “2” in Figure 3 for high- and low-nFLH waters, respectively, their MODISA and VIIRS Rrs spectra appeared similar in both the spectral shapes and magnitudes in the blue-green wavelengths. Even though there was some cross-sensor difference in the blue-green bands, cross-location difference for each sensor was rather small.

Thus, it would be difficult to differentiate the high- and low-nFLH waters using the blue-green bands of either MODISA or VIIRS. In contrast, the Rrs spectra in the red wavelengths showed a local peak at 678-nm from the high-nFLH waters. Such a local peak forms the basis of the 3-band subtraction FLH algorithm [8] (Figure 5) although more sophisticated algorithms have also been developed [25]. Indeed, the nFLH value from Location 1 of Figure 3 was about eight times of nFLH from Location 2 (0.04 *vs.* 0.005 mW·cm^−2^·μm^−1^·sr^−1^), indicating the MODIS capacity in detecting phytoplankton changes in CDOM-rich waters.

Evaluation of MODIS and VIIRS from other dates showed the same results. nFLH imagery revealed spatial gradient within the dark water patches while the default Chla imagery showed more homogeneous patterns. In the absence of shallow-bottom contamination or non-living particles such as suspended sediments, the high nFLH signals within the dark water can only be due to phytoplankton blooms [9,10]. The question is whether these blooms are due to *K. brevis* or other phytoplankton species, which can be confirmed by field measurements.

**Figure 6.**
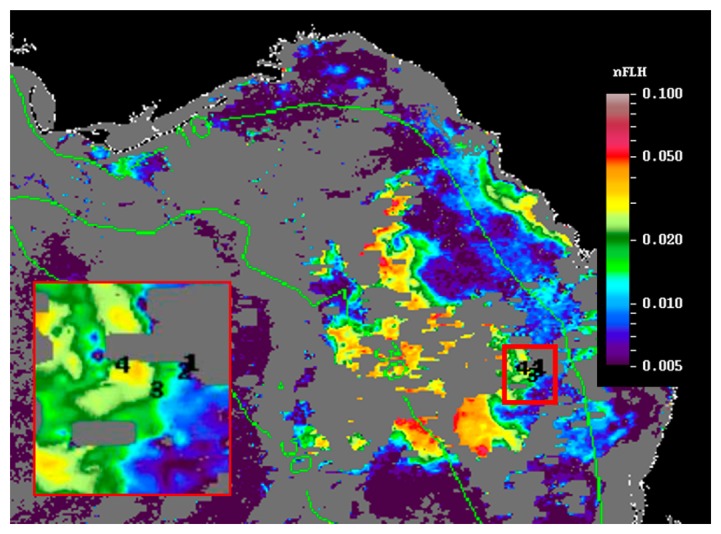
MODISA nFLH image on 23 July 2014 (19:05 GMT) showing an extensive bloom patch of about 7000 km^2^. The corresponding VIIRS ERGB image from an adjacent day (18 July 2014) is shown in Figure 2d. Around the east edge of the bloom 4 stations are annotated (red box, inset figure). The high nFLH values along the coast are likely due to sediment resuspension. Vertical profiles of ocean properties at the four stations, along with *K. brevis* concentrations in surface waters, are shown in Figure 7.

**Figure 7.**
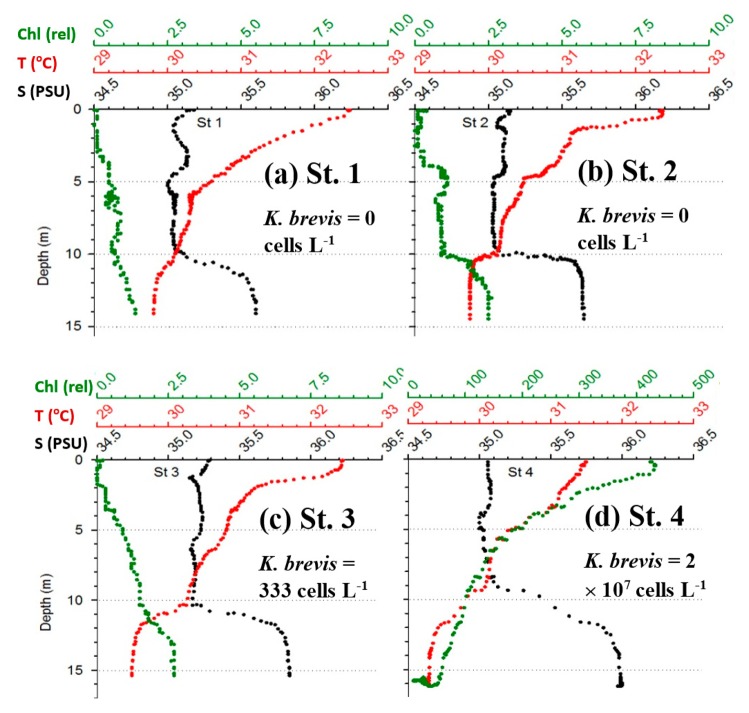
(**a**)–(**d**): Vertical profiles of salinity, temperature, and Chla fluorescence (relative units) at the four sampling stations shown in Figure 6. Note the dramatic difference in their Chla profiles regardless of the similarity in salinity. *K. brevis* concentrations of the surface waters are annotated on each panel.

**Figure 8.**
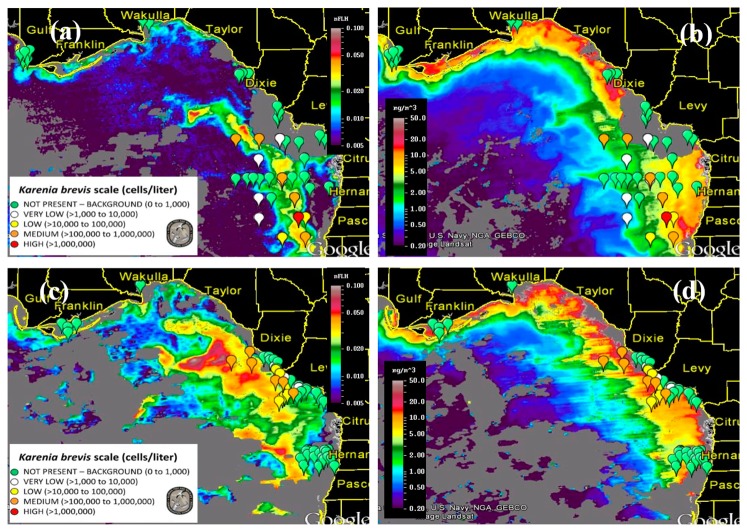
MODISA nFLH and Chla images on 2 September 2014 (**a**,**b**) and on 14 September 2014 (**c**,**d**), annotated with *K. brevis* concentration data between 27 August and 3 September (**a**,**b**) and between 10 September and 17 September (**c**,**d**). (**a**,**c**) are examples of the FWC HAB bulletins distributed weekly or twice weekly. Gray represents clouds or invalid data. More examples can be found in the Supplemental Materials.

**Figure 9.**
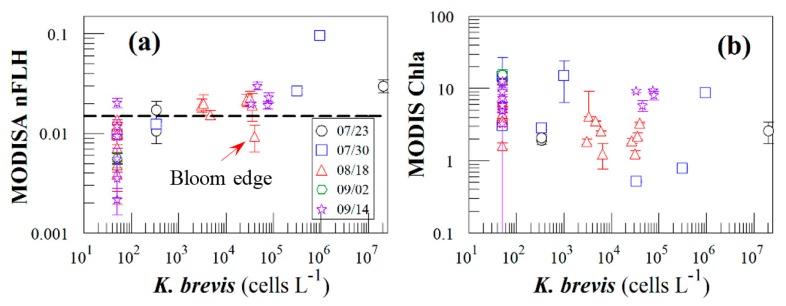
Comparison between MODISA data (nFLH in (**a**) and Chla (in (**b**)) and near-concurrent (±1 day) *K. brevis* concentrations for 6 HAB bulletins on 07/23, 07/30, 08/08, 08/18, 09/02, and 09/14/2014. Data for 08/08 is missing due to cloud cover. The dashed line in (**a**) represents nFLH = 0.015 mW·cm^−2^·μm^−1^·sr^−1^. Chla has units of mg·m^−3^. Concentrations of 0 are artificially lifted to 50 in order to display on the log scale. In (**a**), there are 19 data points with *K. brevis* < 1000 cells·L^−1^ and 14 data points > 1000 cells·L^−1^.

Figure 6 shows the MODIS nFLH image on 23 July 2014 annotated with station locations on the same day. The field samples confirmed the presence of a HAB dominated by *K. brevis* (e.g., 2 × 10^7^ cells·L^−1^ at St. 4, Figure 7d). The combined imagery and field data also showed a tight relationship between MODISA nFLH and *K. brevis* concentration and a poor relationship between MODISA (and VIIRS) Chla and *K. brevis*. The high *K. brevis* concentration at St. 4 corresponded to a high nFLH value of ∼0.03 mW·cm^−2^·μm^−1^·sr^−1^. In contrast, the low nFLH values at Sts. 1–3 all corresponded to 0 to 333 cells·L^−1^ of *K. brevis* (although St. 1 was blocked by clouds, one could infer low nFLH value from cloud-free pixels adjacent to clouds). The data profiles at all four stations showed similar vertical distribution in temperature and salinity but dramatic difference in Chla fluorescence (Figure 7), also confirming bloom waters at St. 4. Note that the 10-m low-salinity surface layer suggested CDOM-rich water, confirming the observations in Figures 2 and 3. Indeed, local river plumes from the Suwannee River and other rivers contain high concentrations of CDOM due to wetland discharges, and CDOM absorption coefficient at 443 nm (*a_g_*443) could be as high as 10 m^−1^ during abnormal discharge periods [26]. Between May and September of a normal discharge year, *a_g_*443 was about 1.6 m^−1^ at salinity = 23.0 PSU, and the *a_g_*443-salinity relationship for salinity > 23 appeared to be dominated by conservative mixing (Figure 4b of [26]). Assuming *a_g_*443 of 0.02 m^−1^ at salinity = 36 PSU (clear ocean water for this region), a conservative mixing would lead to *a_g_*443 of 0.14 m^−1^ at salinity = 35 PSU (the surface layer in Figure 4), representing a dominant component in *a*_t_443 estimated from the dark water patches (∼0.2 m^−1^). Thus, the spatial gradient of *K. brevis* was well revealed by MODIS nFLH but not by VIIRS or MODISA Chla due to the strong perturbations by CDOM. Such observations were confirmed by more concurrent satellite and field data shown in Figures 8 and 9.

Figure 8 presents nFLH and Chla image pairs for 2 days (more image pairs are presented in Supplemental Materials), where weekly *K. brevis* data are annotated on the images. Similar to Figure 6, these images demonstrate that the *K. brevis* spatial patterns are well revealed by the nFLH images, but not by the Chla images. Statistically, Figure 9 shows collocated MODISA (3 × 3 mean) and field data within ±1 day. To assure data quality, only when at least >5 MODISA pixels centered at the station were found to be valid was data extracted to compute the mean. It is clear that compared to Chla, nFLH is a better bloom index (in this case, HAB index) in CDOM-rich waters. If a threshold of nFLH ≥ 0.015 were to be used to detect *K. brevis* blooms (>1000 cells·L^−1^), the false positive rate would be only 1/19 = 5% and the false negative rate would be only 1/14 = 7%. *K. brevis* blooms are known to be very patchy [13] and the false negative point is near the bloom edge, thus not a true failure. Indeed, HAB detection requires human interpretation of the imagery, and from the spatial context (as opposed to a point-point comparison in Figure 9a) the bloom and non-bloom features can be well distinguished from the nFLH imagery with near 100% success (*i.e.*, when the feature was treated as a whole instead of individual pixels, a high-nFLH offshore feature was confirmed to *always* contain high *K. brevis* cells, and low-nFLH waters were confirmed to *always* contain low or zero *K. brevis* cells). In contrast, such fidelity significantly degraded in the Chla imagery in either point-point comparison (Figure 9b) or feature detection (Figure 8). Even the Chla-anomaly imagery, currently used operationally in NOAA's HAB bulletins, did not reveal the bloom patches (results not shown here).

### 3.2. Discussion

The success of using nFLH imagery to detect blooms in CDOM-rich waters is attributed to the fluorescence band, which could also be used in other approaches for bloom detection [14]. Without such a band VIIRS obviously cannot produce nFLH-like imagery. The question is whether VIIRS can differentiate blooms in CDOM-rich waters using other bands. Both QAA and other semi-analytical algorithms [27] were attempted, both yielding unsatisfactory results as they tended to attribute all non-water absorption to CDOM. The default blue-green band ratio Chla algorithm, on the other hand, treated everything as Chla, thus resulting in relatively homogeneous patterns within the dark water. Such a deficiency also creates difficulty in applying the Chla-anomaly based bloom detection method that is being used by NOAA. This deficiency applies to both MODISA and VIIRS because of their low Rrs values from the dark water (Figures 3, 4 and 5). Therefore, at least for this particular event, nFLH provided a better bloom index than Chla and Chla anomaly. Indeed, all field sampling efforts during this event were guided by the nFLH imagery, as indicated by the adaptive sampling locations (e.g., Figures 1, 2, 3, 4, 5, 6, 7 and 8).

Some of the VIIRS sensor artifacts such as banding (e.g., Figure 3d) may be corrected through post processing [28]. On the other hand, some of the small discrepancy between VIIRS and MODIS Rrs spectra in the blue-green wavelengths collected on the same day (and same GMT hour) from the same locations (Figures 4 and 5) could have resulted from the calibration uncertainties, which may be improved in future reprocessing once updated calibration and atmospheric correction algorithms are available. In contrast, the lack of a fluorescence band on VIIRS cannot be overcome through improvement in calibration or atmospheric correction, thus representing a true deficiency of VIIRS in detecting and quantifying coastal blooms in CDOM-rich waters. Such a deficiency can ultimately be removed by future satellite missions with sensors equipped with one or more fluorescence bands, for example the European Space Agency's Sentinel-III Ocean and Land Color Instrument (OLCI, to be launched around June 2015) and NASA's Pre-Aerosol-Cloud-Ecosystem (PACE) mission (currently being planned for launch in 2019) (Bryan Franz, NASA/GSFC, personal communication). Meanwhile improved bio-optical inversion algorithms without using a fluorescence band need to be developed to detect and quantify blooms in dark waters using VIIRS.

However, the case study here should not be over interpreted, as most ocean color data products (e.g., the empirical and semi-analytical *K*_d_ products, the band-ratio and band-subtraction Chla products, the IOP products) are derived without the fluorescence bands. Even for the CDOM-rich waters, VIIRS and MODISA Rrs in the blue-green bands were shown to be very similar (Figures 4 and 5) despite their small discrepancy. They are indeed both effective in detecting and mapping dark waters. Furthermore, whether or not the lack of a fluorescence band limits the VIIRS capacity in studying other types of blooms (e.g., *Alexandrium* sp. in the Gulf of Maine [29]; coccolithophorid blooms in the global ocean [30]) also needs to be studied. As the community is working to improve the calibration and algorithms continuously, it is expected that VIIRS may continue the MODIS observations whenever the use of fluorescence band is not a critical requirement or whenever improved algorithms are developed to detect and quantify blooms in dark waters in the absence of a fluorescence band.

## 4. Conclusions

A harmful algal bloom of *K. brevis* in extensive dark waters of the NEGOM in June–September 2014 was used to compare the capacity of VIIRS and MODISA in mapping dark waters as well as detecting and qualifying the bloom size and intensity. Although both sensors detected the CDOM-rich dark waters in a similar fashion, delineating bloom patches within the dark water currently was only possible with MODISA because of its extra fluorescence band at 678 nm. Because *K. brevis* blooms often form in CDOM-rich dark waters, for effective HABs monitoring, future ocean color sensors should have fluorescence bands, such as those being planned for the Sentinel-III and PACE missions. Meanwhile better algorithms are required for VIIRS to map blooms in CDOM-rich dark waters in the absence of the fluorescence band.

## Supplementary Materials

Supplementary materials can be accessed at: http://www.mdpi.com/1424-8220/15/2/2873/s1.



## Figures and Tables

**Table 1.** MODIS and VIIRS sensor characteristics.

**Sensor**	**Res. (km)**	**Swath**	**Revisit**	Number of Ocean Bands *	**Wavelength Range**	**Duration**
MODISA	1.1 × 1.1	2330 km	1–2 day	9	412–869 nm	2002–present
VIIRS	0.75 × 0.375	3300 km	1 day	7	410–862 nm	2011–present

* Only ocean color bands are listed here. MODISA has two more bands than VIIRS, located at 531 and 678 nm, respectively. The 678-nm MODIS band is used to detect solar stimulated phytoplankton fluorescence, which is not available on VIIRS.
