# Supplementary material for: A Harmful Algal Bloom of Karenia brevis in the Northeastern Gulf of Mexico as Revealed by MODIS and VIIRS: A Comparison
Source: Sensors (Basel). 2015 Jan 28;15(2):2873–87. doi: 10.3390/s150202873 (PMC4367338; doi:10.3390/s150202873)
Supplement: Supplementary file 1 [file sensors-15-02873-s001.pdf]

OPEN ACCESS

sensors

ISSN 1424-8220
www.mdpi.com/journal/sensors

Supplementary Information

A Harmful Algal Bloom of *Karenia brevis* in the Northeastern Gulf of Mexico as Revealed by MODIS and VIIRS: A Comparison. *Sensors* 2015, *15*, 2873-2887

Chuanmin Hu [1,*], Brian B. Barnes [1], Lin Qi [1] and Alina A. Corcoran [2]

[1] College of Marine Science, University of South Florida, St. Petersburg, FL 33701, USA;
E-Mails: bbarnes4@mail.usf.edu (B.B.B.); lqi@mail.usf.edu (L.Q.)

[2] Florida Fish and Wildlife Conservation Commission, Fish and Wildlife Research Institute, St. Petersburg, FL 33701, USA; E-Mail: alina.corcoran@MyFWC.com

* Author to whom correspondence should be addressed; E-Mail: huc@usf.edu;
Tel.: +1-727-5533987.

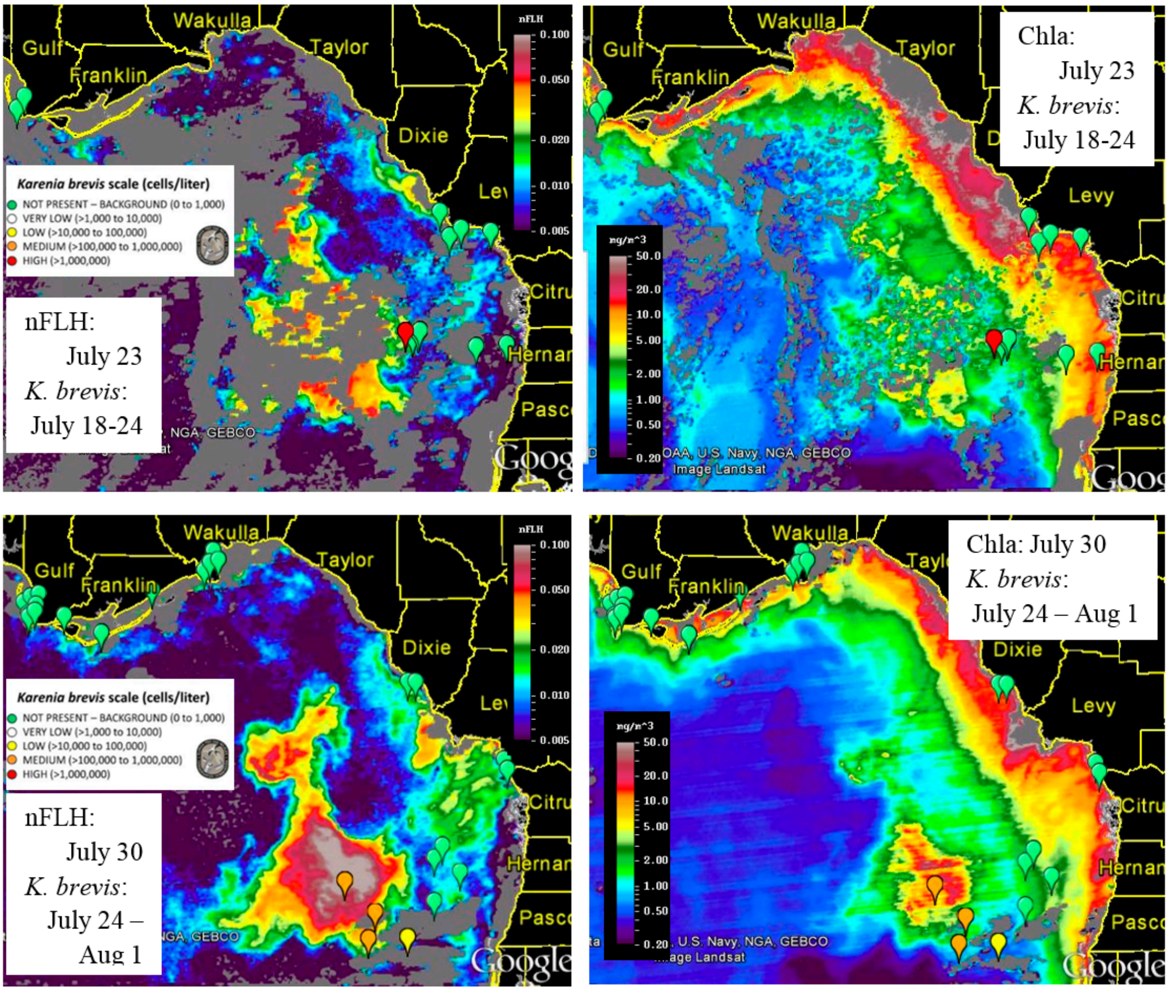

Figure S1. MODISA nFLH and Chla images on 23 July 2014 (**top**) and 30 July 2014 (**bottom**), annotated with *K. brevis* cell counts data between July 18 and July 24 (top) and between July 24 and August 1 (bottom). The left panels are examples of the FWC HAB bulletins distributed weekly or twice weekly. Gray represents clouds or invalid data. All images were generated and distributed routinely in near real-time through a Web interface (http://optics.marine.usf.edu/cgi-bin/optics_data?roi=BIGBEND&current=1).

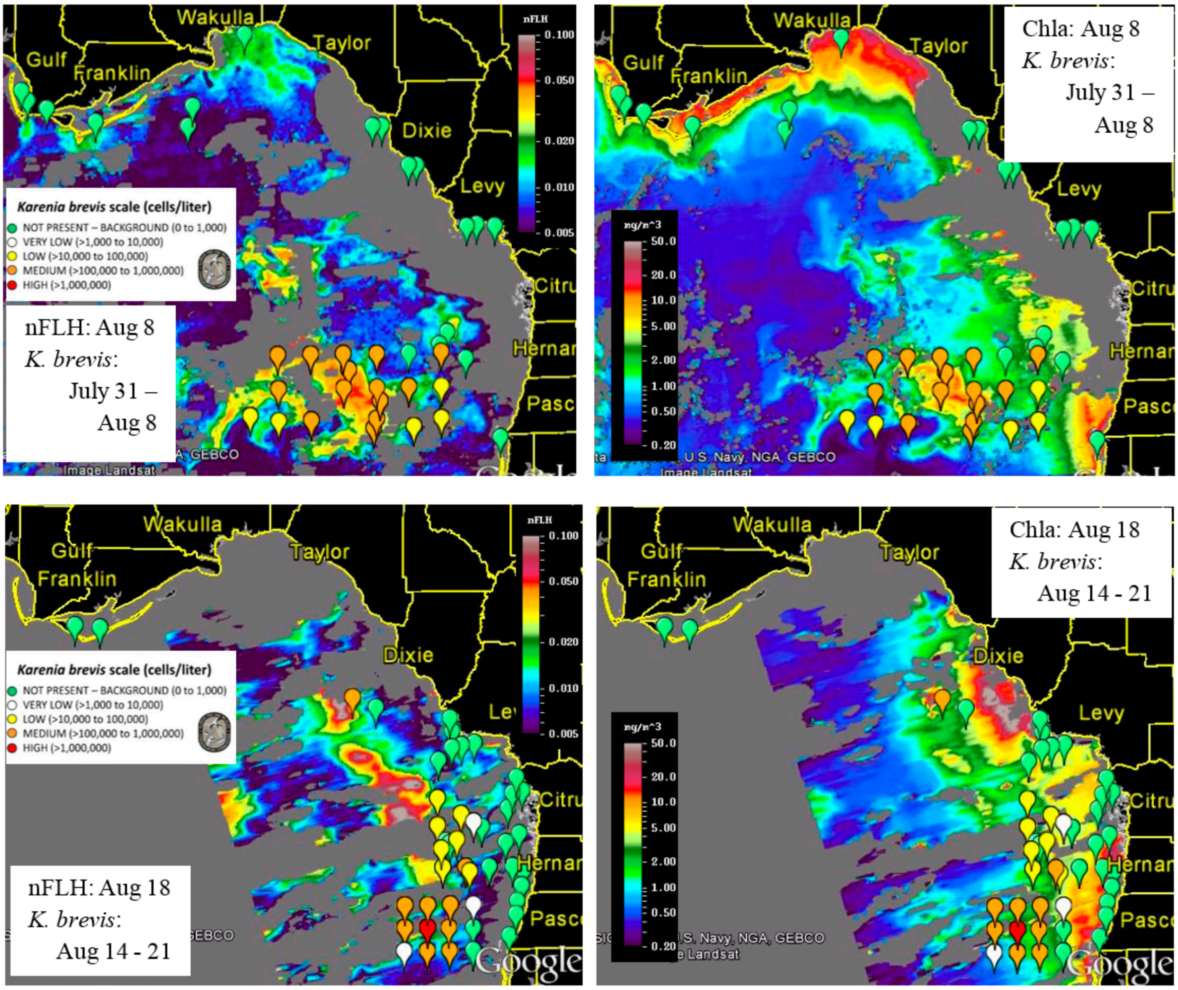

Figure S2. Same as above, but MODISA images are on 8 August 2014 (**top**) and 18 August 2014 (**bottom**). *K. brevis* data were collected between July 31 and August 8 (top) and between August 14 and August 21 (bottom). Note that the bottom images were along the satellite scan edge (*i.e.*, view angles > 60°). Even though, the nFLH image appears to be able to review the *K. brevis* bloom.

© 2015 by the authors; licensee MDPI, Basel, Switzerland. This article is an open access article distributed under the terms and conditions of the Creative Commons Attribution license (http://creativecommons.org/licenses/by/4.0/).
